# Two New Chalcid Wasps (Hymenoptera: Eulophidae and Megastigmidae) Are Parasitoids of *Ophelimus bipolaris* (Hymenoptera: Eulophidae) on *Eucalyptus* in China

**DOI:** 10.3390/insects17050449

**Published:** 2026-04-24

**Authors:** Jin-Bo Sun, Guo-Bao Qin, Jian-Zhong Ning, Yan Qin, Jun Li, Zoya Yefremova, Xia-Lin Zheng

**Affiliations:** 1 Guangxi Key Laboratory of Agro-Environment and Agric-Products Safety, College of Agriculture, Guangxi University, Nanning 530004, China; jepson0205@163.com (J.-B.S.); 15807884553@163.com (G.-B.Q.); 15873796096@163.com (J.-Z.N.); lijunlijun1981@163.com (J.L.); 2 Guangxi Zhuang Autonomous Region State-Owned Qipo Forest Farm, Nanning 530031, China; gxqinyan@163.com; 3 The Steinhardt Museum of Natural History, Israel National Center for Biodiversity Studies and Department of Zoology, Tel Aviv University, Tel Aviv 6997801, Israel

**Keywords:** Chalcidoidea, Eulophidae, Megastigmidae, Opheliminae, molecular phylogeny, *28S* gene, integrative taxonomy

## Abstract

Two new species, *Aprostocetus eucalyptus* Zheng & Yefremova sp. nov. (Hymenoptera: Eulophidae) and *Megastigmus bipolaris* Zheng & Yefremova sp. nov. (Hymenoptera: Megastigmidae), were discovered on populations of the invasive gall wasp *Ophelimus bipolaris* (Hymenoptera: Eulophidae) infesting *Eucalyptus* in Guangxi, China. An integrative taxonomic approach combining morphological characterization and *28S* rRNA-based phylogenetic analysis was used for species identification and classification. Detailed morphological descriptions, illustrations, and an identification key for both sexes are provided. Field parasitism data confirm their potential as native natural enemies, supporting their utility for the biological control of this economically important pest.

## Simple Summary

Two new parasitoid species, *Aprostocetus eucalyptus* sp. nov. (Hymenoptera: Eulophidae) and *Megastigmus bipolaris* sp. nov. (Hymenoptera: Megastigmidae), were discovered in Guangxi, China, parasitizing the invasive gall-forming pest *Ophelimus bipolaris* on *Eucalyptus*. Based on morphological characterization and *28S* rRNA-based molecular evidence, we provided detailed descriptions, illustrations, and an identification key for both sexes of the two species. Their potential for the biological control of this invasive *Eucalyptus* pest was also discussed.

## 1. Introduction

Gall-forming insects are a major threat to *Eucalyptus* plantations globally, with the genus *Ophelimus* (Hymenoptera: Eulophidae) standing out as one of the most destructive invasive groups [1,2]. Native to Australia, these wasps have invaded continents across the world via human-mediated dispersal, inducing abnormal galls on various *Eucalyptus* species [3,4]. The galls disrupt plant nutrient transport, reduce photosynthetic capacity, cause extensive defoliation, and suppress tree growth, ultimately leading to substantial economic losses in infested areas [3]. To date, several prominent *Ophelimus* species, e.g., *O.maskelli* (Ashmead) and *O.eucalypti* (Gahan), have established widespread invasive populations, inflicting severe damage on *Eucalyptus* ecosystems and industries worldwide [5,6].

*Ophelimus bipolaris* Chen & Yao, first reported in China in 2021, is a newly emerging invasive gall wasp that specifically targets hybrid *Eucalyptus* (*Eucalyptus grandis* × *E. urophylla*) [4]. Its rapid expansion and high infestation intensity have posed a serious threat to local *Eucalyptus* plantations, calling for urgent and effective control measures. Biological control using native or co-evolved parasitoids is recognized as a sustainable strategy to manage the invasive *Eucalyptus* gall wasp [7,8,9]. Notably, recent taxonomic studies have documented specialized parasitoids associated with *O. bipolaris* in China, including a new *Aprostocetus* species and *Chrysonotomyia ophelimi* sp. nov., highlighting the potential of hymenopteran parasitoids as targeted biocontrol agents for this invasive gall wasp [10,11].

The genus *Aprostocetus* (Hymenoptera: Eulophidae) comprises over 800 described species worldwide, many of which are primary parasitoids of gall-forming insects, including cynipids, cecidomyiids, and other eulophids [12,13]. Species such as *Aprostocetus causalis* La Salle & Wu have been found to exhibit potential for biological control of the eucalyptus gall wasp *Leptocybe invasa* Fisher & La Salle (Hymenoptera: Eulophidae), parasitizing its mature larvae and pupae [14]. Its parasitism rates vary across regions in China, ranging from 2.3% to 26.3%, and can reach up to ~57.1% when combined with other parasitoids [15]. For the newly recorded *Aprostocetus bipolaris* Zheng & Yefremova, field investigations have confirmed a parasitism rate of 18.52% on its specific host *O. bipolaris*, reflecting its inherent capacity to suppress populations of this invasive gall wasp [11]. Another species, *Aprostocetus gala* (Walker), acts as a parasitoid of *L. invasa* in India and associates with gall midges on multiple plant species, demonstrating its potential as a biocontrol agent [16].

*Megastigmus* (Hymenoptera: Megastigmidae) includes more than 150 species globally, with some of its members parasitizing gall-forming hymenopterans and dipterans [17,18,19]. Parasitic species of *Megastigmus* have been widely used to control insect pests. For example, *Megastigmus brevivalvus* Girault for biocontrol of *Bruchophagus fellis* Girault, which makes galls on *Citrus glauca* (Lindl.) Burkill (Sapindales: Rutaceae), grapefruit, and other *Citrus* spp. [20]. Several species of *Megastigmus* are considered as biocontrol agents against *L. invasa* [9]. Notably, their introduction into Israel led to effective control of *L. invasa*, which was the case with *M. lawsoni* Dogănlar and *M. zvimendeli* Dogănlar [8]. Doganalar and Hassan [21] reviewed total of 33 species of *Megastigmus* Dalman 1820 related with *Eucalyptus* spp. and provided an identification key. Overall, these examples underscore the notable biocontrol potential of *Megastigmus* species.

In July and October 2025, during a survey of natural enemies of *O. bipolaris* in Nanning City and Liuzhou City, Guangxi Zhuang Autonomous Region, China, two unknown parasitoid species emerged from mature galls induced by *O. bipolaris* on hybrid *Eucalyptus*. Preliminary morphological observations suggested that they are species of the *Aprostocetus* and *Megastigmus* genera. To confirm their taxonomic status, we conducted an integrated study using morphological characterization (including SEM and light microscopy) and molecular phylogenetic analyses based on *28S* gene sequences. Our aim is to identify these parasitoid species and evaluate their potential as biological control agents against *O. bipolaris*. Here, we describe these two new species as *Aprostocetus eucalyptus* sp. nov. and *Megastigmus bipolaris* sp. nov. and discuss their potential roles in the biological control of *O. bipolaris*.

## 2. Materials and Methods

### 2.1. Insect Sampling

In July 2025, branches infested with mature galls induced by *O. bipolaris* were collected from Qipo Forest Farm (108°04′35″ E, 22°36′46″ N) in Nanning City. In October 2025, the same host materials were collected from Sanmenjiang Forest Farm (109°42′12″ E, 24°22′10″ N) in Liuzhou City, Guangxi Zhuang Autonomous Region, China. To maintain the branches’ turgor and freshness, the collected materials were placed in a cylindrical plastic container (height × diameter = 15 cm × 13 cm) filled with deionized water and subsequently transferred to a sealed net cage (100 mesh) with dimensions of 40 cm × 40 cm × 80 cm (length × width × height).

For adult eclosion, the branches were incubated at temperature of 26 ± 1 °C, a relative humidity of 70–80%, and a photoperiod of 13 h light: 11 h dark. The mesh cage was examined daily, and newly emerged adults resting on the inner wall of the cage were individually collected using 1.5 mL sterile centrifuge tubes. All adult specimens were temporarily preserved in the centrifuge tubes prior to being euthanized in 75% (*v*/*v*) ethanol and subsequently processed for morphological characterization and molecular identification. Holotypes and paratypes of the two new species were deposited in the College of Agriculture, Guangxi University (GXU).

### 2.2. Morphology

Morphological terminology follows Burks et al. [22]. The following acronyms are applied to morphology: fu_1_–fu_7_, funicular segments 1–7; clv_1_–clv_3_, claval segments 1–3; smv, submarginal vein; mv, marginal vein; pmv, postmarginal vein; stv, stigmal vein; POL, postocellar line; OOL, ocellocular line; msc, mesoscutum; Gt_1_–Gt_7_, gastral tergites 1–7.

Body, ovipositor, and genitalia lengths were measured absolutely (μm), whereas relative measurements were used for other dimensions.

Specimens were identified and photographed with a Keyence VHX-6000 (Keyence, Osaka, Japan) digital microscope and a scanning electron microscope (FEI Quattro S, Thermo Fisher Scientific, Brno, Czech Republic). Final images were processed in Adobe Photoshop 2018 software.

### 2.3. DNA Extraction, Amplification, and Sequencing

Genomic DNA was isolated from adult individuals using the TIANamp Genomic DNA Kit (DP304-02, TianGen, Beijing, China) in strict accordance with the manufacturer’s instructions. For molecular characterization, the *28S* rRNA gene was chosen for amplification, with the specific primer pair D2-3551F (5′-CGTGTTGCTTGATAGTGCAGC-3′) and D2-4057R (5′-TCAAGACGGGTCCTGAAAAGT-3′) [23]. All PCR amplifications were performed on a T100™ Thermal Cycler (Bio-Rad Laboratories Pty. Ltd., Singapore) in a total volume of 40 μL, which consisted of 20 μL premix Taq polymerase (RR902A, Takara, Dalian, China), 16 μL ddH_2_O, 1 μL of each primer (10 pmol/μL), and 2 μL of DNA template (50 ng/μL). The thermal cycling program was set as follows: an initial denaturation step at 94 °C for 3 min; followed by 35 cycles of 94 °C for 30 s, 53 °C for 30 s, and 72 °C for 1 min; with a final extension at 72 °C for 5 min. The quality and fragment size of the resulting PCR amplicons were verified by 1.0% agarose gel electrophoresis, and the qualified products were subjected to bidirectional Sanger sequencing by Sangon Biotech Co., Ltd. (Shanghai, China).

### 2.4. Sequence Alignments and Phylogenetic Analysis

Sequences generated from sequencing were assembled and subjected to preliminary analysis using DNAMAN V6 software. Following quality assessment, all consensus sequences were deposited in the GenBank database (http://www.ncbi.nlm.nih.gov, accessed on 19 August 2025) to acquire unique accession numbers. Homology searches for related sequences were then conducted via the NCBI Nucleotide BLAST tool (version 2.16.0; https://blast.ncbi.nlm.nih.gov/Blast.cgi, accessed on 23 August 2025).

Multiple sequence alignments of the nucleotide data were performed using ClustalW V2 [24]. Phylogenetic analyses were carried out in MEGA V7 [25], with maximum likelihood (ML) phylogenetic trees reconstructed using 1000 bootstrap replicates to assess node support [26].

## 3. Results

### 3.1. Taxonomy

Hymenoptera

Eulophidae Westwood, 1829

Tetrastichinae Graham, 1987

*Aprostocetus* Westwood, 1833

**Figure 1:**
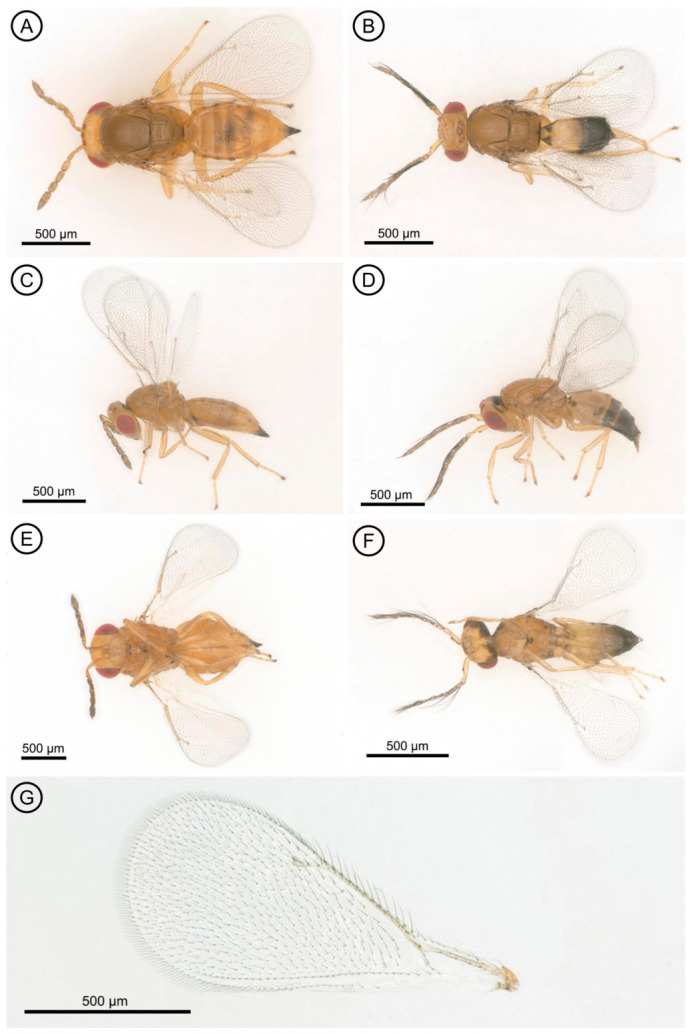
*Aprostocetus eucalyptus*. (**A**) Dorsal view of female; (**B**) Dorsal view of male; (**C**) Lateral view of female; (**D**) Lateral view of male; (**E**) Ventral view of female; (**F**) Ventral view of male; (**G**) Fore wing of female.

**Figure 2:**
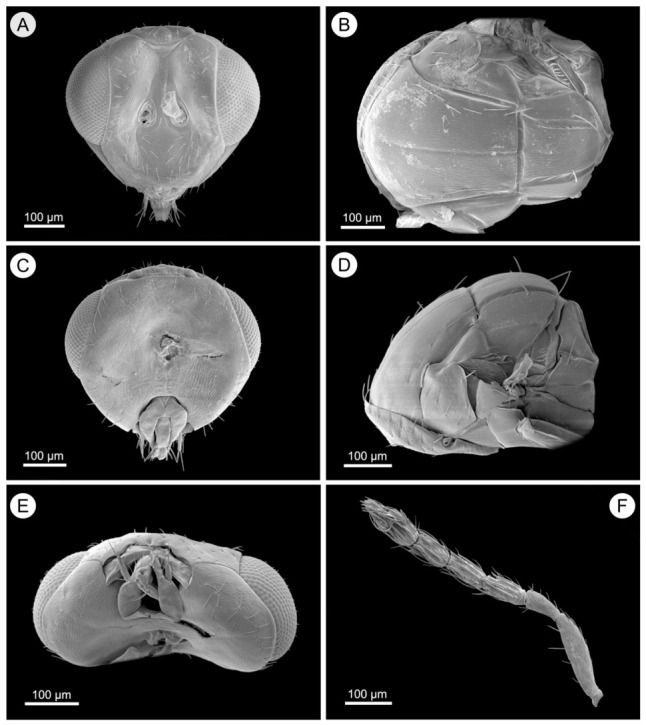
*Aprostocetus eucalyptus* female. (**A**) Frontal view of head; (**B**) Dorsal view of mesosoma; (**C**) Posterior view of head; (**D**) Lateral view of mesosoma; (**E**) Ventral view of head; (**F**) Antenna.

**Figure 3:**
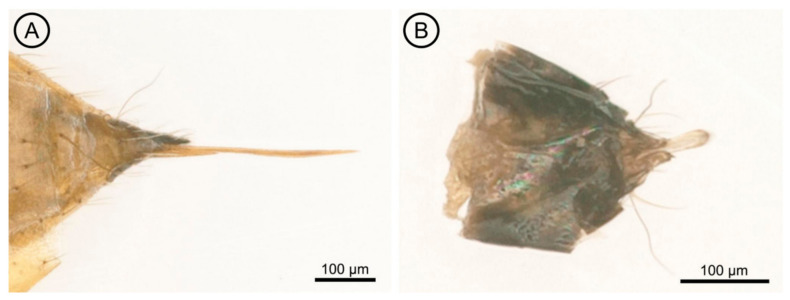
*Aprostocetus eucalyptus*. (**A**) Ovipositor; (**B**) Male genitalia.

**Figure 4:**
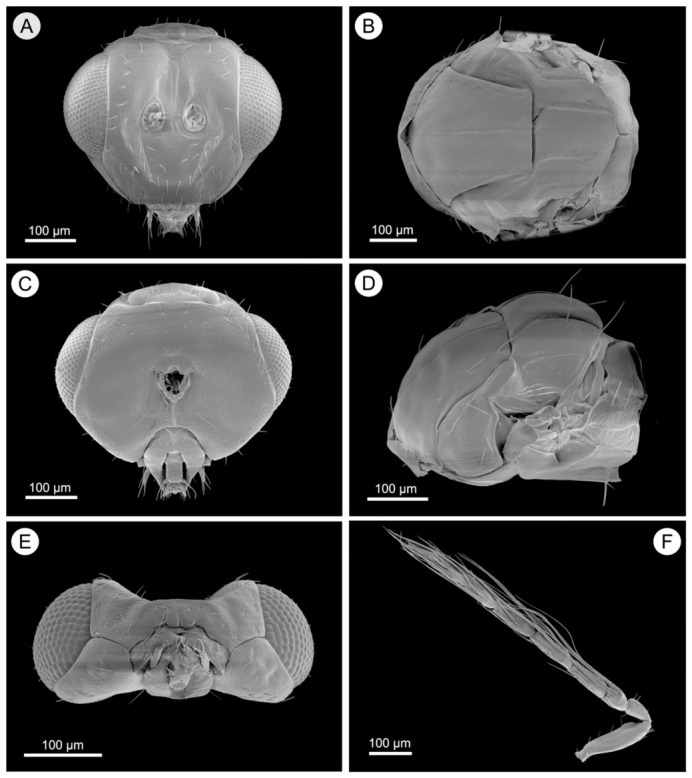
*Aprostocetus eucalyptus* male. (**A**) Frontal view of head; (**B**) Dorsal view of mesosoma; (**C**) Posterior view of head; (**D**) Lateral view of mesosoma; (**E**) Ventral view of head; (**F**) Antenna.

*Aprostocetus eucalyptus* Zheng & Yefremova sp. nov. (Figure 1, Figure 2, Figure 3 and Figure 4).

LSID urn:lsid:zoobank.org:act:E78EA11D-4322-4B81-BEA8-1BAF77A473E3.

Diagnosis. The mesoscutum possesses a distinct median groove, with 5–7 adnotaular setae arranged in a single row along each lateral margin. The propodeal callus bears four setae arranged in two distinct rows. Female: The antenna has a 3.7× longer than wide scape; fu_1_ is 2.0× as long as broad, fu_2_ 1.9× as long as broad, and fu_3_ 1.8× as long as broad. The 3-segmented clava is 2.5× as long as broad and 1.6× the length of fu_3_. The gaster is 1.85× as long as broad. Male: The antenna has a scape 3.6× as long as broad and a ventral plaque accounting for 0.4× the scape length. Funicular segment proportions are as follows: fu_1_ 1.2× as long as broad, fu_2_ 2.7× as long as broad, fu_3_ 3.0× as long as broad, and fu_4_ 2.9× as long as broad. The 3-segmented clava is 3.6× as long as broad. In terms of relative length, fu_1_ is 0.5× that of fu_2_, fu_2_ ≈ 0.83× that of fu_3_, and the clava is 2.5× that of fu_4_. Whorls of fu_1_ reaching tip base of fu_3_. Gaster 2.0× as long as broad. The genitalia, digiti with one developed spine.

Description.

Female: Body length 1.5 mm.

Color (Figure 1A,C,E). Head yellow, vertex laterally brown; eye red; ocelli reddish. Antenna flagellum brown, scape and pedicel yellow. Mesosoma dark brown, dorsellum dark, propodeum yellow. Legs yellow. Gaster yellow with transverse brownish bands on Gt_3_, Gt_4_, Gt_5_ and Gt_6_ (Figure 1A).

Head (Figure 2A,C,E). Measuring 1.3× as long as broad, POL 1.6× the length of OOL. The clypeus is bilobed, and a median longitudinal carina is present on the frons. The mandibles have two large teeth, the eyes are glabrous, and the malar sulcus is straight. The oral opening is 0.77× as wide as the malar space is long. Antenna (Figure 2F) comprises a scape 3.7× as long as broad, a pedicel 2.0× as long as broad, and a single anellus. Funicular segment proportions are as follows: fu_1_ 2.0× as long as broad, fu_2_ 1.9× as long as broad, fu_3_ 1.8× as long as broad. The 3-segmented clava is 2.5× as long as broad, 1.6× the length of fu_3_.

Mesosoma (Figure 2B,D). The mesoscutum is 1.35× as broad as long, with a median line and five adnotaular setae in a single row on each lateral side. The scutellum is 1.5× as broad as long, with two pairs of setae. The distance between the two submedian lines is twice that between the submedian and sublateral lines. The dorsellum is 0.83× the length of the propodeum. The propodeum is 6.0× as broad as long, with rounded spiracles located near its anterior margin and surrounded by a postspiracular rim; its surface is superficially reticulate, and the callus has four setae in two rows. Forewing (Figure 1G) is 2.4× as long as broad, with smv:mv:stv = 24:30:8. mv is as long as the costal cell, and pmv is a short stub. The closed speculum extends to one quarter below mv. smv has three setae, and mv bears 12 setae.

Metasoma. The petiole bears a transverse, small and smooth surface. The gaster (Figure 1A) is 1.85× as long as broad; the cercus has three setae, with the longest seta kinked and 2.5× longer than the next cercal seta (Figure 3A). The ovipositor sheaths (Figure 3A) are slightly extended, with several setae covering their apex.

Male: Body length 1.3 mm.

Color similar to female in general body coloration, except for the gaster, which has a pale yellow basal portion and a darkish brown to black apical half, forming a clear color division (Figure 1B,D,F).

Head (Figure 4A,C,E). Antenna (Figure 4F) with scape 3.6× as long as broad; ventral plaque 0.4× the length of scape, located in upper part. Pedicel 1.9× as long as broad; fu_1_ 1.2× as long as broad, fu_2_ 2.7× as long as broad, fu_3_ 3.0× as long as broad, fu_4_ 2.9× as long as broad; clava 3-segmented 3.6× as long as broad; fu_1_ 0.5× as long as fu_2_, fu_2_ ~0.83× as long as fu_3_. Clava 2.5× as long as fu_4_. Whorls of fu_1_ reaching tip base of fu_3_; Whorls of fu_3_ reaching base of clv_2_; Whorls of fu_4_ reaching base of clv_3_, with whorls of clv_1_ extending.

Mesosoma (Figure 4B,D). Measuring 1.1× as broad as long, the mesoscutum features a distinct median line and five adnotaular setae in a single row on each lateral side. The scutellum is 1.6× as broad as long. The propodeum, which is 7.0× as broad as long, has a superficially reticulate texture, with the callus bearing four setae in the anterior row and two in the posterior row.

Metasoma. The petiole is transverse in shape. The gaster (Figure 1B) is 2.0× as long as broad. The cercus has two dorsal setae, the longest of which is kinked and 1.7× longer than the other cercal seta (Figure 3B). The genitalia is only partially exserted (Figure 3B). Parameres positioned on either side of the aedeagus each have two setae (Figure 3B), and the digiti each possess a single well-developed spine.

Material. Holotype: ♀, China: Nanning City, Qipo Forest Farm (108°04′35″ E, 22°36′46″ N, alt. 160 m), reared from *O. bipolaris* on *E. urophylla* × *E. grandis*, 9-VII-2025, leg. J.-B. Sun, G.-B. Qin & J.-Z. Ning.

Paratypes: 10 ♀ and 2 ♂, same data as holotype (deposited in GXU, preserved in 75% ethanol).

Distribution. China (Guangxi).

Etymology. The species is named after its host plant *Eucalyptus* (the host of *O. bipolaris*). The Chinese name for it is 桉树叶疱长尾啮小蜂 (eucalyptus torymid wasps).

Comments. Comparison with *A. bipolaris* Zheng & Yefremova, 2025

1. Female antenna with scape 4.0× as long as broad and clava 2.0× as long as broad. Gaster 1.6× as long as broad. Male antenna with ventral plaque 0.33× the length of scape; clava 2.4× as long as fu_4_ and 5.6× as long as broad. Whorls of fu_1_ reaching ⅓ basal part of fu_2_. Body dark brown with green tint; vertex metallic green. Parasitoid of *Ophelimus bipolaris*......................................................................................................................................................*A. bipolaris* Zheng & Yefremova

- Female antenna with scape 3.7× as long as broad and clava 2.5× as long as broad. Gaster 1.85× as long as broad. Male antenna with ventral plaque 0.4× the length of scape; clava 2.5× as long as fu_4_ and 3.6× as long as broad. Whorls of fu_1_ reaching tip of fu_3_. Body brownish without metallic tint; vertex yellow. Parasitoid of *Ophelimus bipolaris*.........................................................................................................................................................................*A. eucalyptus* sp. nov.

Hymenoptera

Megastigmidae Janšta et al., 2018 [27]

Megastigminae Thomson, 1876

*Megastigmus* Dalman, 1820

**Figure 5:**
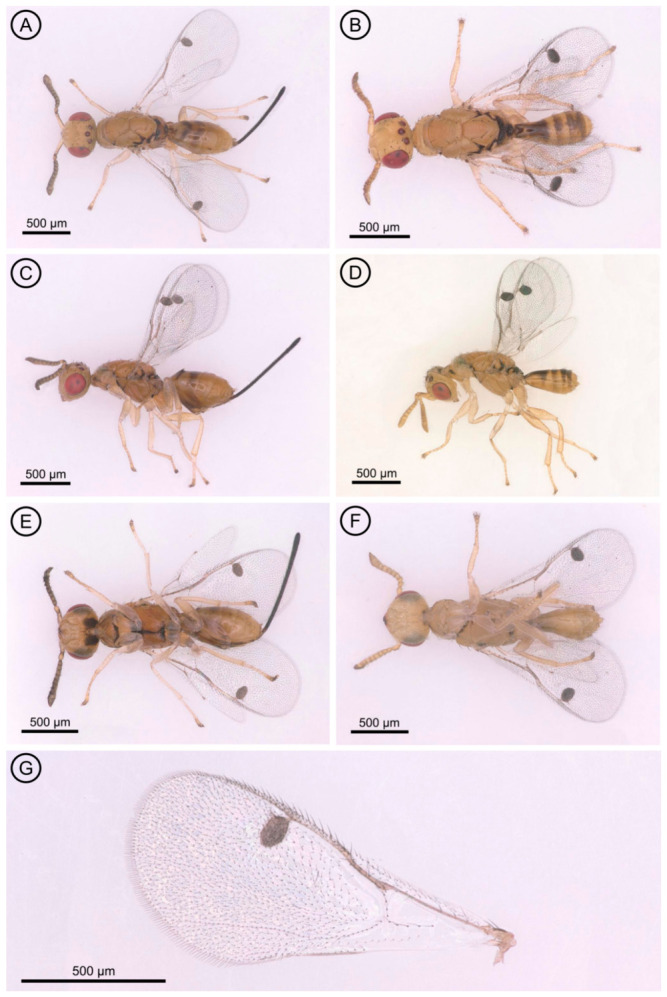
*Megastigmus bipolaris*. (**A**) Dorsal view of female; (**B**) Dorsal view of male; (**C**) Lateral view of female; (**D**) Lateral view of male; (**E**) Ventral view of female; (**F**) Ventral view of male; (**G**) Fore wing of female.

**Figure 6:**
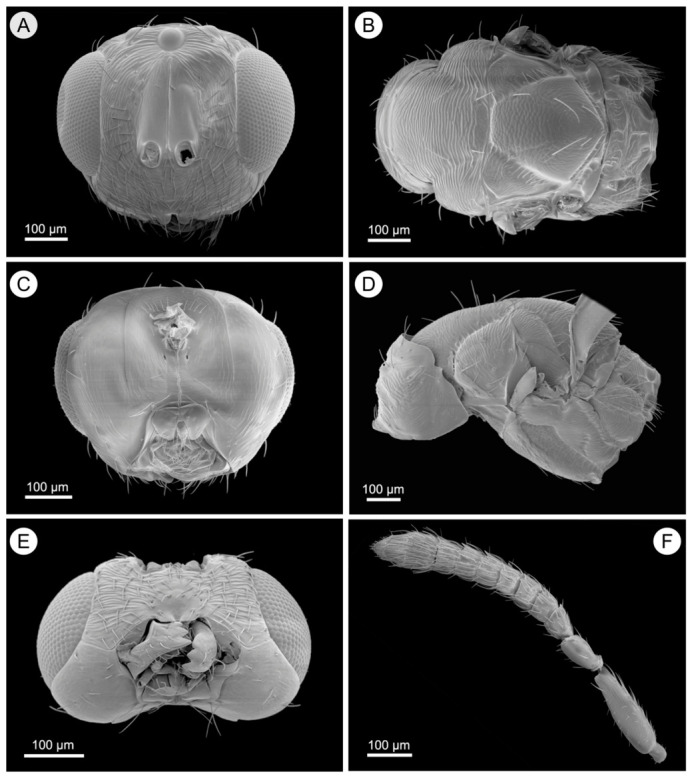
*Megastigmus bipolaris* female. (**A**) Frontal view of head; (**B**) Dorsal view of mesosoma; (**C**) Posterior view of head; (**D**) Lateral view of mesosoma; (**E**) Ventral view of head; (**F**) Antenna.

**Figure 7:**
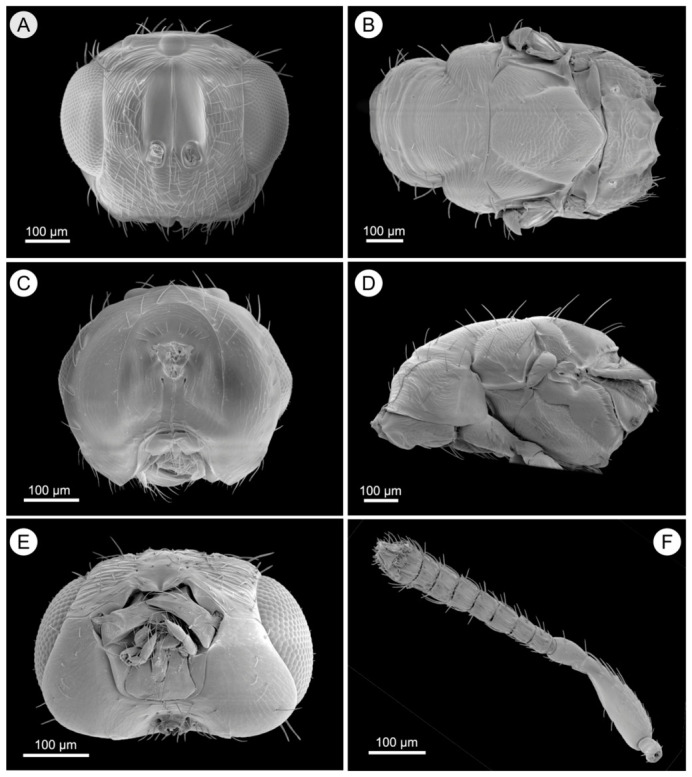
*Megastigmus bipolaris* male. (**A**) Frontal view of head; (**B**) Dorsal view of mesosoma; (**C**) Posterior view of head; (**D**) Lateral view of mesosoma; (**E**) Ventral view of head; (**F**) Antenna.

*Megastigmus bipolaris* Zheng & Yefremova sp. nov. (Figure 5, Figure 6 and Figure 7).

LSID urn:lsid:zoobank.org:act:DE420136-F29D-41A2-967F-2A92192A010A.

Diagnosis. Female. Antenna: all funicular segments transverse; length of clava equal to length 2.8 preceding segments. Scutellum with 3 pairs of setae; frenal groove distinct, frenum crenulated. Propodeum with median carina, which is interrupted in ⅓ anterior part by transverse carina; plicae present, and the area between spiracles with superficial reticulation. Gaster 2.2× as long as wide. Sheaths of ovipositor 1.45× as long as gaster. Ovipositor 0.55× as long as body, 1.4× as long as metasoma, 2.6× as long as hind tibia. Male. Antenna: scape cylindrical, 2.1× as long as broad; funicular subquadrate, two last funiculars transverse. Length of clava equal to that of the preceding 2.5 segments. Propodeum with indistinct median carina and interrupted in ⅓ anterior part by transverse carina; plicae present. Gaster 2.6× as long as wide. Body yellow, pilosity black.

Description.

Female. Body size (without ovipositor) 1.66 mm; length of the exerted part of sheaths 0.83 mm.

Color (Figure 5A,C,E). Body yellow. Head with yellow vertex. Face yellow. Eyes and ocelli red. Mesosoma yellow; anterior margin of propodeum brown. Gaster yellow, with Gt_1_, Gt_3_ and Gt_5_ brown. Wings hyaline; veins yellow; parastigma and stigma dark brown. Legs yellow. Pilosity of body black.

Head (Figure 6A,C,E). Width 1.3× as long as height and 1.5× as long as length. POL 2.0× as long as OOL. Antennae inserted slightly above the lower margin of eyes. Relative measurements. Antenna (Figure 6F): scape (60 × 20), pedicel (29 × 15); one anellus; fu_1_ (15 × 17), fu_2_ (14 × 18), fu_3_ (15 × 18), fu_4_ (14 × 18), fu_5_ (14 × 20), fu_6_ (14 × 20), fu_7_ (14 × 20); all funiculars transverse; clava (40 × 24).

Mesosoma (Figure 6B,D). Pronotum 3.5× as broad as long, with superficial reticulation; midlobe of mesoscutum with scapulae 1.5× as broad as long, finely transversely striate; scutellum 1.1× as long as broad, with superficial reticulation and three pairs of setae, frenal groove distinct, frenum crenulated; propodeum 3.2× as broad as long, propodeal callus with numerous setae in three rows. Propodeum 1.1× as long as scutellum; median carina present, interrupted in ⅓ anterior part by transverse carina; plicae present, with the area between spiracles bearing superficial reticulation; spiracles with rim. Fore wing 2.5× as long as broad. Relative measurements of forewing: smv:mv (including parastigma):pmv:stv (including stigma) = 47:53:34:17. So, pmv 2.0× as long as stv. Stigma 1.7–1.8× as long as width (Figure 5G). Costal cell at most 13× as long as its maximum width, hairy; basal cell open and bare; speculum open and broad. Parastigma 0.4× as long as mv; area below mv narrow on both sides, without row of admarginal setae; area between pmv and uncus narrow, with two setae on upper side, bare on under side.

Metasoma. Petiole short and transverse. Gaster (Figure 5A) 2.2× as long as wide. Sheaths of ovipositor 0.7× as long as body. Sheaths of ovipositor 1.45× as long as gaster. Ovipositor 0.55× as long as body, 1.4× as long as metasoma, 2.6× as long as hind tibia.

Male. Body size 1.8 mm.

Color similar to female except as follows: body darker; propodeum fully dark brown; gaster with Gt_1_, Gt_2_, Gt_5_, Gt_7_ dark brown (Figure 5B,D,F).

Head (Figure 7A,C,E). POL 1.5× as long as OOL. Antenna (Figure 7F): scape (75 × 26), pedicel (28 × 18); one anellus; fu_1_ (15 × 15), fu_2_ (17 × 17), fu_3_ (17 × 18), fu_4_ (17 × 18), fu_5_ (17 × 18), fu_6_ (16 × 18), fu_7_ (14 × 20); two last funiculars transverse; clava (60 × 26).

Mesosoma (Figure 7B,D). Pronotum 4.2× as broad as long; midlobe of mesoscutum 2.0× as broad as long; scutellum 1.5× as long as broad. Pronotum and msc finely transversely striate. Scutellum with 3 pairs of setae and with superficial reticulation. Propodeum 3.4× as broad as long; propodeal callus with numerous setae in three rows; median carina indistinct, interrupted in ⅓ anterior part by transverse carina; plicae present, with the area between spiracles bearing superficial reticulation; spiracle with rim. Fore wing 2.1× as long as broad; smv with 6 setae.

Metasoma. Petiole short; gaster (Figure 5B) (65 × 25), 2.6× as long as wide.

Material. Holotype: ♀, China: Nanning City, Qipo Forest Farm (108°04′35″ E, 22°36′46″ N, alt. 160 m), reared from *O. bipolaris* on *E. urophylla* × *E. grandis*, 9-VII-2025, leg. J.-B. Sun, G.-B. Qin & J.-Z. Ning.

Paratypes: 276 ♀ and 247 ♂, China: Guangxi Zhuang Autonomous Region, Liuzhou City, Sanmenjiang Forest Farm (109°42′12″ E, 24°22′10″ N, alt. 311 m), reared from the same host and plant, 15-X-2025, leg. J.-B. Sun & Y. Qin (deposited in GXU, preserved in 75% ethanol).

Distribution. China (Guangxi).

Etymology. The species is named after its host, *O. bipolaris*. The Chinese name for it is 桉树叶疱大痣小蜂 (eucalyptus megastigmine wasps).

Identification

In the key to the Palearctic species of *Megastigmus* native and introduced to the West Palearctic region [28], *M. bipolaris* sp. nov. (female) runs to couplet 8, which should be modified as follows:

8. Thorax mostly brownish black, except pronotum more or less yellowish; funicular segments subquadrate [28] (p. 150); pronotum more than 2.0× wider than long; frenal area smooth, without longitudinal carinae [28] (p. 193); forewing stigma oblong, 1.4× as long as broad, with a very long uncus about half as long as stigma length [28] (p. 164). Body 1.8 mm. Host: seeds of *Sorbus* spp. and *Amelanchier* spp. (Rosaceae)....................................................................................*M. brevicaudis* Ratzeburg

8a. Thorax mostly yellow including pronotum; funicular segments transverse (Figure 5A); pronotum more than 3.0× wider than long; frenal area reticulate, with longitudinal carinae (Figure 5C); forewing stigma oblong, 1.8× as long as broad, with uncus about ¼ as long as stigma length (Figure 5G). Body 1.66 mm. Host: galls of *Ophelimus bipolaris* on *Eucalyptus*....................................................................................................................................................................*M. bipolaris* sp. nov.

In the key to the species of *Megastigmus* Dalman, 1820 reared from galls of *Leptocybe invasa* Fisher & La salle, 2004 [29], based on the characters of body, except forewing, *M. bipolaris* sp. nov. (female) runs to couplet 8, which should be modified as follows:

8. Ovipositor 0.62× as long as body, 1.3× as long as metasoma, 3.5× as long as hind tibia; propodeum without median carina; metasoma 1.4× as long as mesosoma; antenna with 1-st funicular segment 2.0× as long as wide. Body 1.5–2.1 mm. Hosts: gall of *Leptocybe invasa* on *Eucalyptus*...................................................................................................*M. thailandiensis* Doğanlar & Hassan

8a. Ovipositor 0.55× as long as body, 1.4× as long as metasoma, 2.6× as long as hind tibia; propodeum with median carina; metasoma as long as mesosoma; antenna (Figure 6F) with with 1-st funicular segment 1.02× as wide as long. Body 1.66 mm. Host: galls of *Ophelimus bipolaris* on *Eucalyptus*......................................................................................................*M. bipolaris* sp. nov.

In the key to the species of *Megastigmus* (33 species) associated with *Eucalyptus* spp. in Australia [21], *M. bipolaris* sp. nov. (female) runs to couplet 46(45), which should be modified as follows:

46(45′). Mesosoma 2.3× as long as mesoscutum broad; pronotum about 1.6× as broad as long. Metasoma 0.7× as long as mesosoma. Body 2.0 + 1.2 mm. Thailand, 2001 from galls of *Leptocybe invasa* on *Eucalyptus*…...........................................................................................................................….*M. thitipornae* Doğanlar & Hassan
46a(45′). Mesosoma 1.76× as long as mesoscutum broad; pronotum about 3.5× as broad as long. Metasoma as long as mesosoma. Body 1.66 + 0.83 mm. China, 2025 from galls of *Ophelimus bipolaris* on *Eucalyptus*..........................................................................................................................................................*M. bipolaris* sp. nov.

*Megastigmus bipolaris* sp. nov. (male) runs to couplet 53(52), which should be modified as follows:

52. Scutellum with 3 pairs of setae..............................................................................................................................................................53
53(52). Scape 1.3× as long as transfer diameter of eye; club as long as 3 preceding segments combined; metasoma 1.4× as long as hind tibia and 0.7× as long as mesosoma.................................................................................*M. thitipornae* Doğanlar & Hassan
53a(52). Scape 1.25× as long as transfer diameter of eye; club as long as 2.5 preceding segments combined; metasoma 1.66× as long as hind tibia and as long as mesosoma..................................................................................................*M. bipolaris* sp. nov.

### 3.2. Phylogenetic Analyses

The *28S* rRNA gene was successfully amplified using PCR. The amplified products from *A. eucalyptus* sp. nov. and *M. bipolaris* sp. nov. specimen were 583 bp and 610 bp. These obtained sequences have been submitted to the NCBI database (*A. eucalyptus*: PX212766; *M. bipolaris*: PX212767). NCBI Nucleotide Blast showed that *Aprostocetus* sp. (PV911623) exhibited the highest sequence similarity (99.33%) to the target sequences of *A. eucalyptus* sp. nov., and *Megastigmus* sp. 1 (MT383732) exhibited the highest sequence similarity (100.00%) to the target sequences of *M. bipolaris* sp. nov. Complete pairwise genetic distance matrices for all analyzed *Aprostocetus* and *Megastigmus* species are provided in Supplementary Tables S1 and S2, respectively.

**Figure 8:**
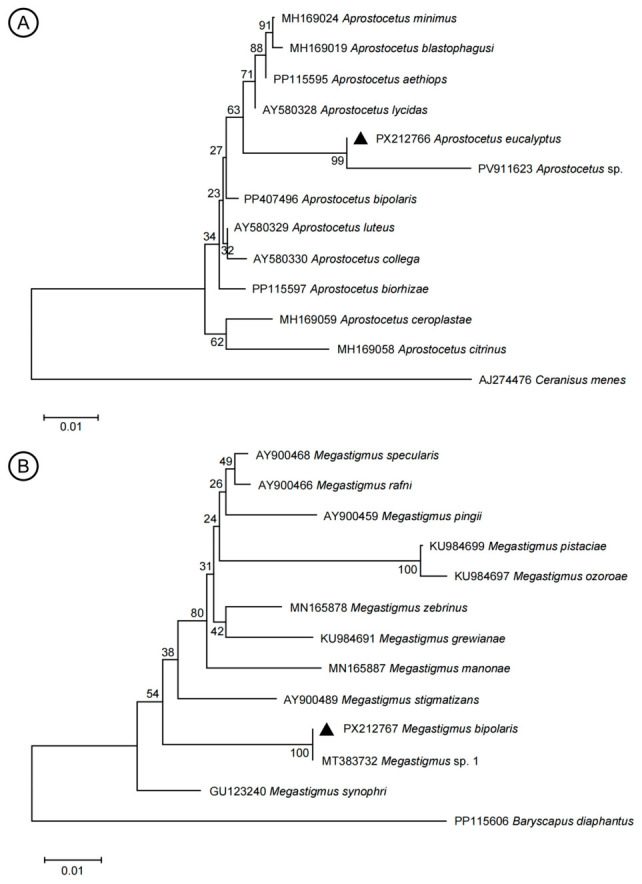
Maximum likelihood trees based on *28S* rRNA sequences. (**A**) *Aprostocetus eucalyptus* sp. nov. (black triangle); (**B**) *Megastigmus bipolaris* sp. nov. (black triangle). The number at each branch indicates the percentage supported by bootstrap. The scale bar indicates substitutions per site.

For the target gene sequence of *A. eucalyptus* sp. nov. in this study, phylogenetic analysis clarified its phylogenetic placement as follows (Figure 8A): *A. eucalyptus* sp. nov. formed a subclade with *Aprostocetus* sp. (PV911623). This subclade was further clustered with the lineage containing *Aprostocetus lycidas* (AY580328), and these combined lineages were nested within a larger monophyletic clade composed of multiple *Aprostocetus* species. This topological structure indicates a close evolutionary relationship between *A. eucalyptus* sp. nov. and other representatives of the genus *Aprostocetus*. In addition, the subclade harboring *A. eucalyptus* sp. nov. shows clear divergence from other subclades within the *Aprostocetus* clade, suggesting that *A. eucalyptus* sp. nov. has genetic distinctness relative to the known congeneric species included in this phylogenetic framework. The genetic distance between *A. eucalyptus* sp. nov. and *Aprostocetus* sp. (PV911623) is 1.6%, and between *A. eucalyptus* sp. nov. and other analyzed *Aprostocetus* species ranges from 1.8% to 11.6% (Supplementary Table S1). These phylogenetic findings collectively confirm that *A. eucalyptus* sp. nov. belongs to the genus *Aprostocetus*, exhibiting close evolutionary affinity with congeneric species while being genetically differentiated from the reported *Aprostocetus* taxa analyzed herein.

For the target gene sequence corresponding to *M. bipolaris* sp. nov. in this study, phylogenetic analysis results demonstrated the following (Figure 8B): *M. bipolaris* sp. nov. clustered with *Megastigmus* sp. 1 (MT383732) into an independent subclade, indicating an extremely close phylogenetic relationship between these two taxa. Meanwhile, this subclade was nested within the same major clade together with multiple *Megastigmus* species, which suggests a close evolutionary affinity between *M. bipolaris* sp. nov. and members of the genus *Megastigmus*. Furthermore, the subclade comprising *M. bipolaris* sp. nov. and *Megastigmus* sp. 1 (MT383732) showed distinct genetic differentiation from other subclades of *Megastigmus* species within the same major clade, implying a certain genetic uniqueness of *M. bipolaris* sp. nov. relative to the known species in this genus. The genetic distance between *M. bipolaris* sp. nov. and *Megastigmus* sp. 1 (MT383732) is 0.0%, and between *M. bipolaris* sp. nov. and other analyzed *Megastigmus* species ranges from 3.7% to 11.8% (Supplementary Table S2). These phylogenetic findings confirm that *M. bipolaris* sp. nov. in this study is a member of the genus *Megastigmus* and is genetically distinct from other reported congeneric species.

### 3.3. Biology and Biological Control Potential

*Aprostocetus eucalyptus* sp. nov. and *Megastigmus bipolaris* sp. nov. were reared from galls of *O. bipolaris* on *E. urophylla* × *E. grandis* in Guangxi, China. At Qipo Forest Farm in July 2025, adults of *A. eucalyptus* sp. nov. (n = 107) showed a male-biased sex ratio (♀:♂ = 1:1.18; 49♀/58♂) and a parasitism rate of 5.84%. Adults of *M. bipolaris* sp. nov. (n = 196) from the same site showed a female-biased sex ratio (♀:♂ = 1:0.83; 107♀/89♂) and a parasitism rate of 10.7%. At Sanmenjiang Forest Farm in October 2025, adults of *M. bipolaris* sp. nov. (n = 225) showed a female-biased sex ratio (♀:♂ = 1:0.76; 128♀/97♂) and a higher parasitism rate of 14.49%. These parasitism rates suggest potential biocontrol utility against *O. bipolaris*.

## 4. Discussion

Morphological analyses confirmed the distinctiveness of both new species within their respective genera. For *A. eucalyptus* sp. nov., key diagnostic traits such as specific setation patterns on the mesoscutum and propodeum and sexual dimorphism in antennae structure (e.g., female scape 3.7× as long as broad, male ventral plaque 0.4× the length of the scape) clearly differentiate it from congeneric species like *A. bipolaris* [11] and *A. causalis* [14]. Similarly, *M. bipolaris* sp. nov. is distinguished by unique morphological features including transverse funicular segments in females, a distinctively crenulated frenum, and an ovipositor 0.55× the body length—traits that separate it from closely related taxa such as *M. thailandiensis* [21] and *M. brevicaudis* [28]. These morphological characters align with the diagnostic frameworks of the genera *Aprostocetus* and *Megastigmus*, confirming the placement of the new species within these lineages.

Molecular phylogenetic analyses based on *28S* rRNA gene sequences further validated the taxonomic status of the new species. *Aprostocetus eucalyptus* sp. nov. formed a well-supported subclade with *Aprostocetus lycidas* (AY580328), with a genetic distance of 1.8%, and other congeneric species, exhibiting 99.33% sequence similarity to *Aprostocetus* sp. (PV911623), a specimen isolate collected from Lohaghat, Uttarakhand, India, corresponding to a genetic distance of 1.6%. Such a high degree of sequence similarity strongly indicates that these two isolates may represent conspecific populations. Notably, no formal morphological description, voucher specimen information, or definitive host association records have been published for the Indian isolate to date, with genetic distances ranging from 1.8% to 11.6% from other analyzed congeneric species, while showing clear genetic divergence from other reported *Aprostocetus* taxa. This result is consistent with the utility of *28S* rRNA in resolving phylogenetic relationships within Eulophidae [30,31]. For *M. bipolaris* sp. nov., the 100% sequence similarity to *Megastigmus* sp. 1 (MT383732) from Conjola, New South Wales, Australia, which was reared from galls on *Eucalyptus* sp. [19], corresponding to a genetic distance of 0.0%, and clustering within the *Megastigmus* genus clade confirm its affinity to this group, and the complete sequence identity of the *28S* rRNA gene confirms an extremely close phylogenetic affinity and potential conspecificity. However, definitive taxonomic resolution requires a direct morphological comparison with authentic voucher specimens from the type locality in Australia, with genetic distances ranging from 3.7% to 11.8% from other analyzed *Megastigmus* species, while distinct genetic differentiation from other subclades highlights its uniqueness. The congruence between morphological and molecular data underscores the robustness of integrative taxonomy in resolving species boundaries, particularly for small, morphologically conserved parasitoid wasps [32].

The discovery of *A. eucalyptus* sp. nov. and *M. bipolaris* sp. nov. holds significant implications for the biological control of *O. bipolaris*. As an invasive pest with expanding distribution in southern China, *O. bipolaris* currently lacks effective and environmentally friendly management strategies, with chemical insecticides offering limited success due to the protected gall-forming larvae [7]. Parasitoid wasps are among the most effective biocontrol agents for gall-forming insects, owing to their high host specificity and ability to target concealed larvae [33]. Collectively, these findings indicate that these two parasitoid lineages may possess a broader transcontinental distribution range than previously recognized, which is likely associated with the global anthropogenic dispersal of *Eucalyptus* plantations and their co-evolved gall-forming hymenopteran pests. Congeners of the new species, such as *A. causalis*, have been successfully deployed against *Eucalyptus* gall wasps like *Leptocybe invasa* [14], while *Megastigmus* species have been documented as natural enemies of gall-forming hymenopterans [34]. This suggests that *A. eucalyptus* sp. nov. and *M. bipolaris* sp. nov. may exhibit similar biocontrol potential, warranting further investigation into their life history and field adaptability to provide theoretical basis and biological materials for the sustainable management of *O. bipolaris* in China.

## Data Availability

The original contributions presented in the study are included in the article; further inquiries can be directed to the corresponding authors.

## Supplementary Materials

The following supporting information can be downloaded at: https://www.mdpi.com/article/10.3390/insects17050449/s1, Table S1. Interspecific analysis of genetic distance values based on *28S* sequences between various species of *Aprostocetus* analyzed in this study. Table S2. Interspecific analysis of genetic distance values based on *28S* sequences between various species of *Megastigmus* analyzed in this study.
